# Spatial and temporal effects of cortical cerebral microinfarcts on the cortical and subcortical regions in cerebral small vessel disease

**DOI:** 10.1002/alz.71056

**Published:** 2025-12-29

**Authors:** Hao Li, Annemieke ter Telgte, Marco Duering, David G Norris, José P. Marques, Frank‐Erik de Leeuw, Anil M. Tuladhar

**Affiliations:** ^1^ Department of Neurology, Donders Center for Medical Neurosciences Radboud University Medical Center Nijmegen The Netherlands; ^2^ VASCage‐Centre on Clinical Stroke Research Innsbruck Austria; ^3^ Department of Neurology Medical University of Innsbruck Innsbruck Austria; ^4^ Medical Image Analysis Center (MIAC AG) and Department of Biomedical Engineering University of Basel Basel Switzerland; ^5^ Institute for Stroke and Dementia Research (ISD) LMU University Hospital LMU Munich Germany; ^6^ Cognition and Behaviour, Center for Cognitive Neuroimaging Radboud University, Donders Institute for Brain Nijmegen The Netherlands

**Keywords:** cerebral small vessel disease, cortical cerebral microinfarcts, quantitative MRI, tractography

## Abstract

**INTRODUCTION:**

We assessed cortical thickness and micro‐structural abnormalities associated with cortical cerebral microinfarcts (CMIs) and their temporal changes in cerebral small vessel disease (SVD).

**METHODS:**

Fifty‐four participants with SVD underwent monthly magnetic resonance imaging for 10 months. Recent and old CMIs and mirrored contralateral controls were identified. Cortical and subcortical expansions around these regions were used to extract cortical thickness, R1, and neurite density index (NDI).

**RESULTS:**

In cortex, both recent and old CMIs showed altered NDI and R1 values only in immediate perilesional regions, likely reflecting partial‐volume effects. In subcortex, recent CMIs showed no differences from controls, whereas old CMIs exhibited lower NDI along white matter tracts extending ≈10 mm. Longitudinally, recent CMIs showed acute R1 reduction and NDI increase at the lesion site, which normalized on follow‐up.

**DISCUSSION:**

Old CMIs are associated with substantial subcortical microstructural injury along tracts but minimal cortical involvement, whereas alterations in recent CMIs are localized and transient.

**Highlights:**

- This is the first study to comprehensively assess both spatial (cortical and subcortical) and temporal effects of microstructural changes associated with cortical microinfarcts (CMIs) in cerebral small vessel disease using quantitative multi‐modal MRI.
- We employed multiple advanced imaging measures, including cortical thickness, R1 (1/T1) mapping, and neurite densitivity index (NDI) derived from neurite orientation dispersion and density imaging (NODDI) modeling to sensitively detect tissue microstructural alterations.
- We found that old CMIs were associated with pronounced subcortical microstructural injury along white matter tracts but showed minimal cortical involvement, whereas recent CMIs presented localized and transient changes.

## 1 BACKGROUND

Cortical cerebral microinfarcts (CMIs) are small ischemic lesions frequently observed through microscopic neuropathological examination.^1^ Larger CMIs, up to 4 mm in diameter, can be detected in vivo through magnetic resonance imaging (MRI), appearing as hypointense on T1‐weighted images (reflecting old lesions), or hyperintense on diffusion‐weighted imaging (DWI; reflecting recent lesions).^1^ The presence of CMIs has been associated with cognitive impairment, particularly in cerebrovascular disease and dementia populations.^2^, ^3^, ^4^ This association could be attributed partially to cortical atrophy and disruption of white matter (WM) connectivity related to these lesions.^4^, ^5^, ^6^, ^7^ However, the mechanisms by which CMIs lead to cortical and subcortical structural damage remain unclear.

Secondary neurodegeneration is proposed as a potential mechanism through which CMIs contribute to widespread injury,^8^ whereby CMI‐induced damage may propagate in two ways: (1) horizontally within the cortex and (2) vertically toward subcortical regions along descending fibers. Previous neuroimaging studies in humans have shown perilesional cortical atrophy beyond the CMI lesion core^5^, ^6^; although mice models indicate that microinfarct‐related injury may spread along WM tracts.^9^ These findings provide partial support for the hypothesis of secondary neurodegeneration; however, direct evidence in humans, particularly regarding subcortical propagation, is still limited. Moreover, the specific microstructural changes accompanying these processes remain poorly characterized.

Quantitative MRI techniques such as R1 (1/T1) mapping and neurite orientation dispersion and density imaging (NODDI) based on biophysical diffusion modeling provide sensitive markers of microstructural changes.^10^, ^11^, ^12^ R1 primarily reflects myelin content and tissue water, whereas the NODDI‐derived neurite density index (NDI) quantifies axon and dendrite density. Combining R1 and NDI offers complementary insights into tissue microstructure, enabling a comprehensive assessment of cortical and subcortical alterations associated with CMIs.

In addition to spatial propagation, the temporal evolution of CMI‐induced abnormalities may reveal their long‐term consequences. In our previous study with 10 monthly MRI scans, we detected recent CMIs in 13% of participants with small vessel disease (SVD), which later disappeared in follow‐up scans.^13^ However, whether such transient lesions also lead to lasting cortical thinning or microstructural disruption remains unknown.

Here, we aimed to systematically characterize both the spatial and temporal patterns of CMI‐induced tissue damages in SVD patients using multi‐modal quantitative MRI. Spatially, we assessed cortical and subcortical changes surrounding recent and old CMIs. Temporally, we examined longitudinal microstructural changes exclusively for recent CMIs. We hypothesized that (1) CMI‐related abnormalities extend beyond the lesion core into adjacent cortex and subcortical regions along WM tracts, and (2) for recent CMIs, these lesion‐induced changes persist over time, even after the lesions are no longer visible on follow‐up scans.

## 2 METHODS

### 2.1 Participants and MRI acquisition

**FIGURE 1:**
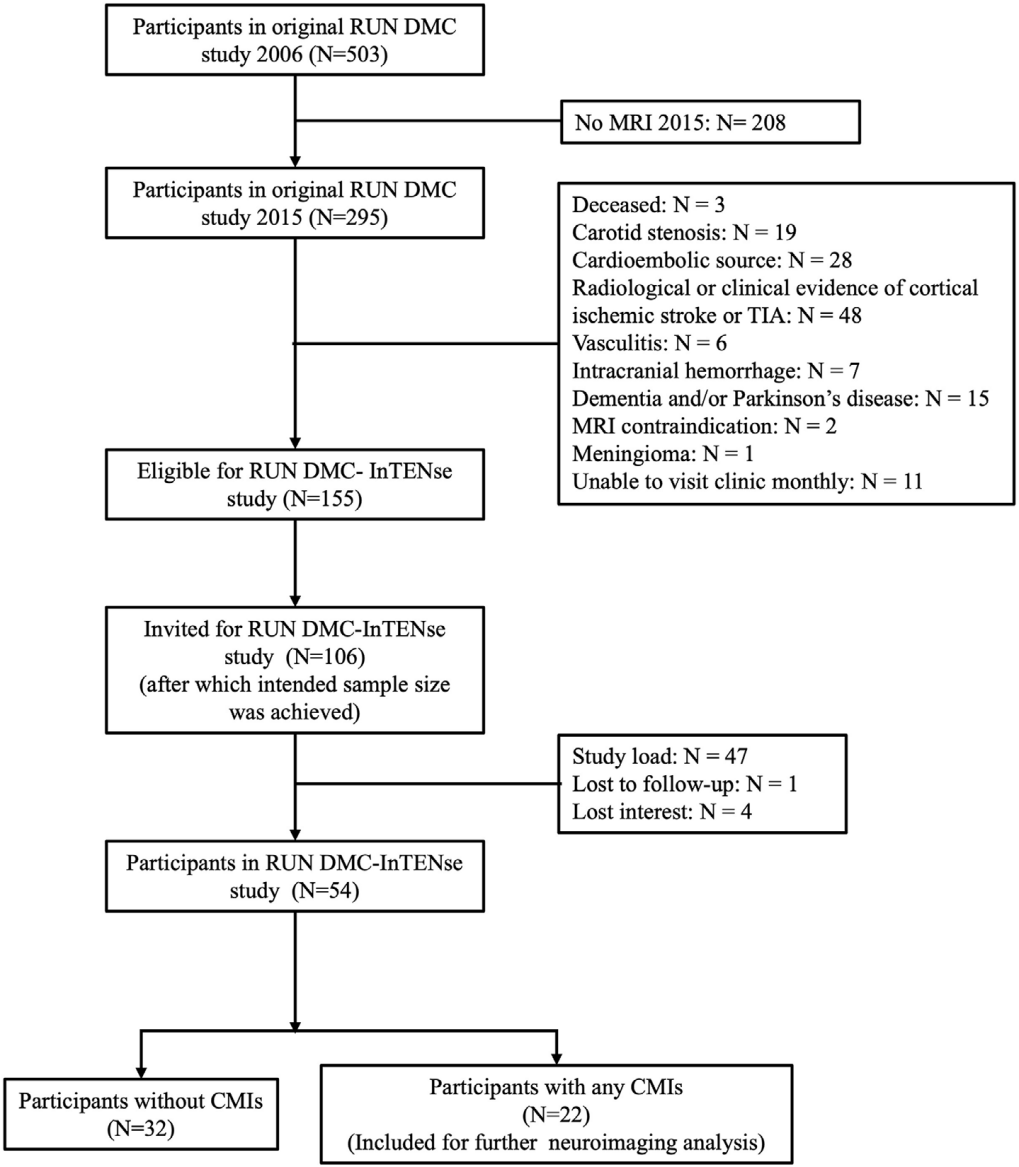
Flow diagram of the study sample. CMI, cortical cerebral microinfarct; RUN DMC, the Radboud University Nijmegen Diffusion tensor and Magnetic resonance imaging Cohort; RUN DMC–InTENse, the Radboud University Nijmegen Diffusion tensor and Magnetic resonance imaging Cohort – Investigating The origin and evolution of cerebral small vessel disease.

**FIGURE 2:**
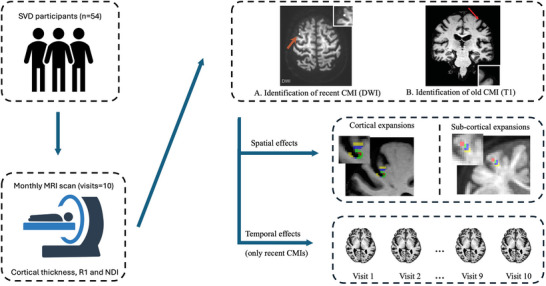
Study design and methods. Participants with SVD underwent 10 consecutive monthly MRI assessments. Cortical thickness, R1, and NDI were quantified from the collected T1 and diffusion MRI. Both recent and old CMIs were identified and segmented from diffusion‐weighted and T1‐weighted images for each participant. The effects of these CMIs on cortical thickness, R1, and NDI were examined both temporally and spatially. Spatial effects were assessed in two complementary ways: (A) cortical expansions, where lesion masks were expanded across the cortical segmentation to evaluate cortical effects; and (B) subcortical expansions, where lesion masks were expanded along reconstructed white matter tracts passing through the lesion to evaluate downstream subcortical effects. Red: lesion site, green: first expansion (1.7 mm), blue: second expansion (3.4 mm), yellow: third expansion (5.1 mm). CMI, cortical cerebral microinfarct; NDI, neurite density index; SVD, small vessel disease.

Data were obtained from the Radboud University Nijmegen Diffusion tensor and Magnetic Resonance imaging Cohort–Investigating the origin and Evolution of Cerebral Small Vessel Disease (RUN DMC–InTENse) study.^14^ RUN DMC‐InTENse is a longitudinal observational study aiming to investigate the origin and short‐term dynamical evolution of SVD. A total of 54 participants with SVD and without other identified causes of cerebral ischemia were recruited from the original RUN DMC study (Figure 1).^13^ All participants underwent a pre‐screening visit to determine eligibility, followed by 10 consecutive monthly MRI assessments at Radboud University Medical Centre between March 2016 and November 2017. A schematic diagram of this study is presented in Figure 2.

MRI scans were performed on a 3T MAGNETOM Prisma scanner (Siemens) with the following sequences: magnetization prepared 2 rapid acquisition gradient echo (MP2RAGE), multi‐shell DWI, three‐dimensional (3D) fluid‐attenuated inversion recovery (FLAIR), multiple spin‐echo T2 image, and 3D multi‐echo gradient echo sequence (GRE). Detailed MRI parameters were described elsewhere.^14^

### 2.2 Identification of cortical cerebral microinfarcts

Assessment of old and recent CMIs has been reported elsewhere.^13^ Briefly, CMIs were defined as MRI‐visible lesions smaller than 4 mm in the cortex. Recent CMIs were hyperintense on DWI, and old CMIs were hypointense on T1W, T2W, and FLAIR images, without hyperintensity on DWI.^1^, ^2^, ^13^, ^15^, ^16^ For the detection of recent CMIs, all monthly images were assessed. Recent CMIs were segmented in DWI space, whereas old CMIs were segmented in T1W space (example images in Figure S1). For each segmented lesion, a corresponding control region was identified by mirroring the lesion mask to the homologous location in the contralateral hemisphere using FMRIB Software Library (FSL), followed by visual inspection and manual adjustment to ensure anatomic correspondence.

**RESEARCH‐IN‐CONTEXT**

- **Systematic review**: The authors conducted a systematic review of the literature using traditional sources (e.g., PubMed). Cortical cerebral microinfarcts (CMIs) have been associated with cognitive impairment in populations with cerebrovascular diseases. This association could be partially attributed to cortical atrophy and the disruption of white matter (WM) connectivity resulting from CMIs. However, the mechanisms by which CMIs lead to cortical atrophy and WM connectivity disruption are not fully understood.
- **Interpretation**: In participants with cerebral small vessel disease (SVD), we found that old CMIs showed lower values of neurite density index (NDI) in the subcortical regions compared to the control regions, but we did not find significant changes in the surrounding cortical regions. In contrast, recent CMIs only showed lower R1 values and higher NDI values locally within the lesion site, which normalized on follow‐up scans. These findings suggest that old CMIs are associated with damage of subcortical regions along the WM tracts, whereas the damage caused by recent CMIs is confined to the lesion site and tend to be transient.
- **Future directions**: Although our study suggests that old CMIs are associated with subcortical WM damage (as measured by low NDI values, indicating axonal damage) along WM tracts. Further histopathological studies are required to validate this finding. In addition, longitudinal studies are needed to explore the temporal relationship between old CMIs and subcortical WM changes.

### 2.3 Quantitative MRI measures: cortical thickness, R1, and neurite density index (NDI)

Three quantitative MRI measures: cortical thickness, R1, and neurite density index (NDI), were employed in our study. Cortical thickness indexes macroscopic morphometric changes. R1 and NDI were chosen to probe underlying microstructural changes. Higher R1 values are generally associated with increased myelination, whereas higher NDI values reflect greater neurite density.^10^, ^11^, ^12^ Considering the potential registration errors between volume space and surface space, these MRI measures were computed directly in the volume space. Cortical thickness and R1 maps were derived from the MP2RAGE sequence (taking into account transmit field inhomogeneities),^17^ whereas NDI maps were generated from fully pre‐processed multi‐shell DWI images using the NODDI toolbox in Matlab.^12^ Note that NDI maps were calculated separately for gray matter (GM) and WM, resulting in two NDI maps (i.e., GM‐NDI and WM‐NDI) for each participant.^18^ Details are provided in the supplementary materials.

### 2.4 Cortical and subcortical expansion

In line with our hypothesis that CMI‐induced damage may propagate along cortical and subcortical pathways, we applied stepwise expansions of the lesion and corresponding control regions in two ways: (1) cortical expansion within adjacent cortex areas in T1 space and (2) subcortical expansion along WM tracts that connect with the lesions or control regions in DWI space. WM tracts connecting with cortical regions of interest (ROIs) were reconstructed using MRtrix 3.0. The initial analyses included three expansion layers for both cortical and subcortical regions. The expansion step size (1.7 mm) corresponded to the voxel resolution of the T1 (0.85 mm) and DWI (1.7 mm) images to minimize interpolation errors. These procedures yielded three concentric rings located ≈1.7 mm, 3.4 mm, and 5.1 mm from the lesion core (example images in Figure S1). Preliminary results indicated that old CMIs showed minimal cortical effects but exhibited consistent subcortical alterations in NDI across the initial three layers. We therefore extended the analysis by increasing the number of sub‐cortical expansion layers from three (5.1 mm) to ten (17 mm). This extended expansion allowed a comprehensive assessment of NDI changes in subcortical regions along WM tracts. Details are provided in the supplementary material.

### 2.5 Extraction of quantitative MRI measures

To assess the cortical spatial effects, we extracted mean cortical thickness, R1, and GM‐NDI values from each cortical expansion of CMIs and control regions; for subcortical spatial effects, we similarly extracted the mean R1 and WM‐NDI values from their subcortical expansions.

To examine the temporal effects of recent CMIs, we defined the image visit at which a lesion was first identified as the “lesion‐visit,” visits prior to this as “pre‐lesion‐visits,” and those after it as “post‐lesion‐visits.” Cortical thickness, R1, and NDI maps from all visits were registered to the T1W image obtained at the lesion‐visit. These MRI measures (cortical thickness, R1, and GM‐NDI) were then extracted from lesion sites and corresponding control ROIs across all visits (further details provided in the supplementary material). To control for global changes unrelated to lesions over time, we calculated the normalized values as ratios of these MRI measures at lesion sites to those in matched control regions.

### 2.6 Statistical analysis

Baseline characteristics of participants with any (recent or old) CMIs were compared to those without using the two‐sample *t*‐test or Mann–Whitney *U* test for continuous variables and the chi‐square test for categorical variables.

First, we tested the spatial (both cortical and subcortical) effects of recent and old CMIs. For cortical expansions, we compared the mean cortical thickness, R1, and GM‐NDI values between lesion expansion and matched control regions. For the initial three subcortical expansions, we compared mean R1 and WM‐NDI values between lesion expansion and control regions. Prior to comparisons, we assessed normality using the Shapiro–Wilk test. We then applied *t*‐tests or Wilcoxon signed‐rank tests depending on whether the data were normally distributed. For extended subcortical expansions, we calculated normalized WM‐NDI values (i.e., lesion/control ratios) and tested whether these normalized values were significantly less than 1 using one‐sample *t*‐tests or Wilcoxon signed‐rank tests as appropriate. To further characterize the spatial gradient of WM‐NDI changes in the sub‐cortical regions, we fitted a linear mixed‐effects model with normalized WM‐NDI values as the dependent variable and expansion distance, lesion size, and their interaction as fixed effects. Random intercepts for each lesion were nested within subjects to account for repeated measurements across lesions and individuals. This model allowed us to assess whether CMI‐related microstructural damage decreased with increasing distance from the lesion and whether this spatial gradient was modulated by lesion size. β values are reported as estimates ± standard error (SE).

Next, to assess the temporal effects of recent CMIs, we first compared the mean cortical thickness, R1, and GM‐NDI values at the recent CMI lesion sites to corresponding controls on lesion‐visit using paired‐sample *t*‐tests (or Wilcoxon signed‐rank tests when normality was not satisfied). Then, for each recent CMI, we averaged these normalized values separately for pre‐lesion and post‐lesion visits. Subsequently, we employed analysis of variance (ANOVA), followed by post hoc paired‐sample *t*‐test or Wilcoxon signed‐rank tests, as appropriate, to compare the normalized values at three time points: pre‐lesion visits, lesion visit, and post‐lesion visits. These analyses focused exclusively on recent CMIs, as old CMIs appear as fluid‐filled cavities on MRI, making assessments of these MRI measures meaningless.

All statistical analyses were performed using R software (version 4.1.1), with alpha set at 0.05 (two‐tailed). Multiple comparisons were corrected using the Bonferroni method.

### 2.7 Sensitivity analysis

Given consistently lower NDI values across three initial subcortical expansions of old CMIs compared to controls, we tested the specificity of WM‐NDI index by comparing mean diffusivity (MD) values between lesion and control regions across the three subcortical expansions using paired‐sample *t*‐tests or Wilcoxon signed‐rank tests as appropriate.

## 3 RESULTS

A total of 54 participants were included (median age 68 years, interquartile range [IQR] 65–73; 63% male). Baseline characteristics comparing participants with any (recent or old) CMIs (*n* = 22) and without (*n* = 32) are presented in Table 1. In total, 472 MRI scans were collected over a median follow‐up of 39.5 weeks for these participants. Across these scans, 21 recent CMIs were identified in 7 participants, and 81 old CMIs were found in 19 participants at baseline; 4 participants exhibited both recent and old CMIs. No additional old CMIs appeared during follow‐up. All recent CMIs were transient, being visible only on the scan at their first appearance and absent on all subsequent MRI visits.

**TABLE 1:** Demographic, clinical, and imaging characteristics of the study sample.

	No CMIs (*n* = 32)	Recent or old CMIs (*n* = 22)	*p* values
**Baseline demographics**			
Age, years, mean (SD)	67.7 (4.0)	73.1 (8.0)	**0.007**
Male, *n* (%)	17 (53.1%)	17 (77.3%)	0.090
Education levels, median (IQR)	5.0 (5.0–6.0)	5.0 (5.0–6.0)	0.628
**Baseline cardiovascular risk factors**			
Systolic blood pressure, mmHg, mean (SD)	138.3 (18.6)	148.9 (19.2)	**0.0496**
Diastolic blood pressure, mmHg, median (IQR)	79.7 (74.2–85.8)	83.3 (77.8–90.6)	0.307
Hypertension, *n* (%)	24 (75.0%)	21 (95.5%)	0.067
Diabetes, *n* (%)	4 (12.5%)	2 (9.1%)	1.000
Hypercholesterolemia, *n* (%)	16 (50.0%)	11 (50.0%)	1.000
BMI, kg/m^2^, mean (SD)	25.7 (4.0)	25.9 (3.5)	0.881
Smoking history, *n* (%)	22 (68.8 %)	16 (72.7%)	0.991
Antithrombotic agents, *n* (%)	13 (40.6%)	13 (59.0%)	0.290
**Baseline SVD MRI markers**			
WMH volume, mL, median (IQR)	3.4 (2.1–9.4)	7.2 (3.9–10.4)	0.138
WMH volume, % of WM volume, median (IQR)	0.8 (0.5–2.0)	1.5 (0.9–8.3)	0.138
Lacunes, *n* (%)	5 (15.6%)	7 (31.8%)	0.194
Microbleeds, *n* (%)	14 (43.8%	11 (50.0%)	0.861

*Note*: Educational level was assessed using a 7‐point Dutch rating scale with 1 indicating primary school not 17 completed and 7 academic degrees. The *p*‐values in bold represent *p* value < 0.05.

Abbreviation: BMI, body mass index; CMIs, cerebral microinfarcts; WM, white matter; WMH, white matter hyperintensity; SVD, small vessel disease.

### 3.1 Cortical spatial effects of CMIs

**FIGURE 3:**
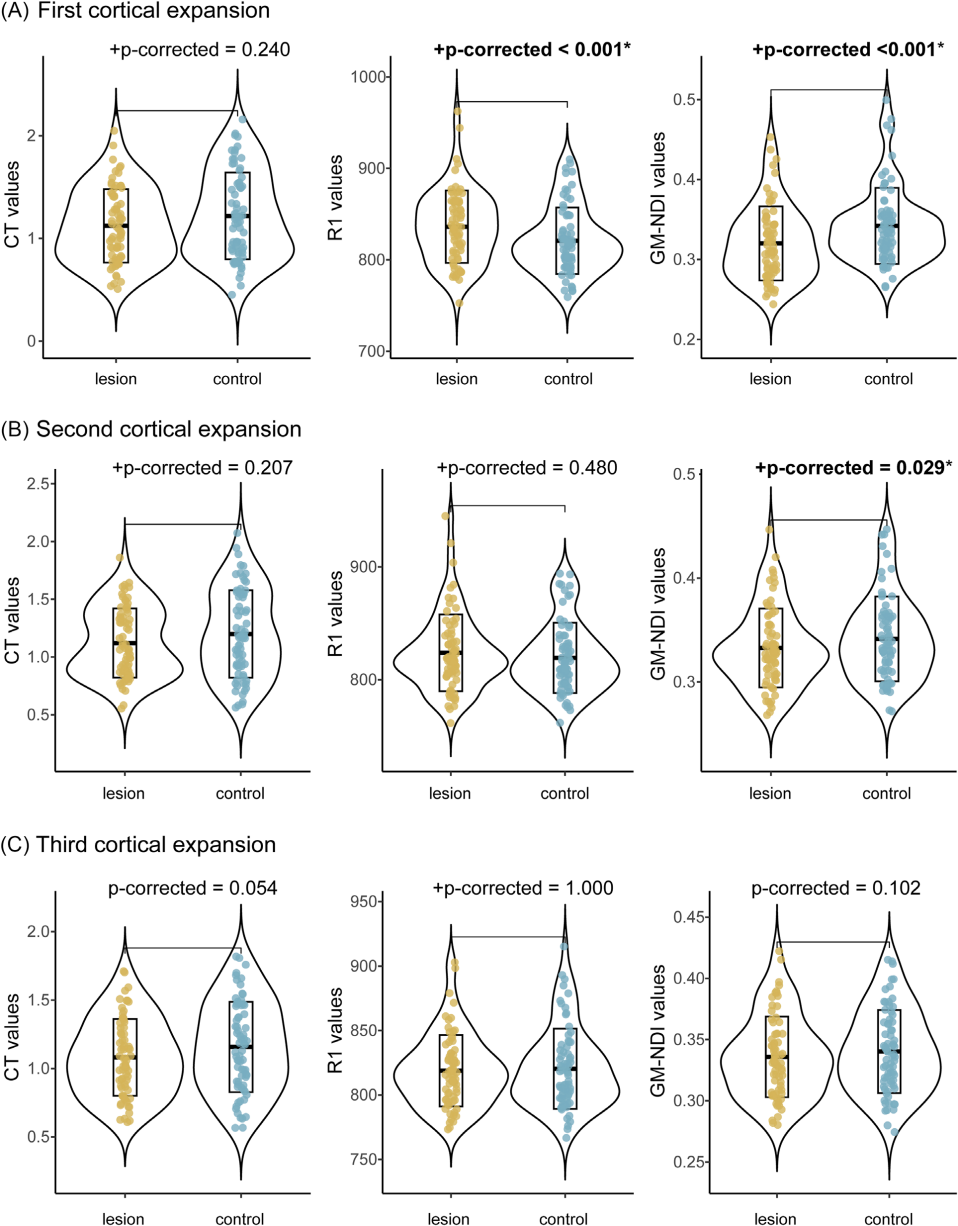
Comparisons of cortical thickness, R1, and GM‐NDI values between old CMIs and control regions at each of their three cortical expansions. Group comparisons were conducted using paired‐sample *t*‐tests or Wilcoxon signed‐rank tests (denoted by “+”), as appropriate. (A) First cortical expansion, old CMIs showed higher R1 values and lower GM‐NDI values compared to control regions. (B) Second cortical expansion, old CMIs showed lower GM‐NDI values compared to control regions. (C) Third cortical expansion, old CMIs no differences at these MRI measures compared to control regions. Significant differences (*p*‐corrected < 0.05) are indicated by an asterisk (*) and bold font. Among the 81 identified old CMIs, seven lesions located too close to the pial surface and two lesions with poor cortical expansion characteristics were excluded, resulting in a total of 72 old CMIs included in the analysis. CMI, cortical cerebral microinfarct; GM‐NDI, gray matter neurite density index.

For recent CMIs, GM‐NDI values were higher in the first lesion expansion compared to the corresponding control regions, with no significant differences at the second or third expansions. (Figure S2). For old CMIs, GM‐NDI values were lower at the first and second lesion expansions compared to corresponding control regions (Figure 3). In addition, for old CMIs, R1 values at the first cortical expansion were higher than those at the corresponding control regions (Figure 3).

### 3.2 Subcortical spatial effects of CMIs

**FIGURE 4:**
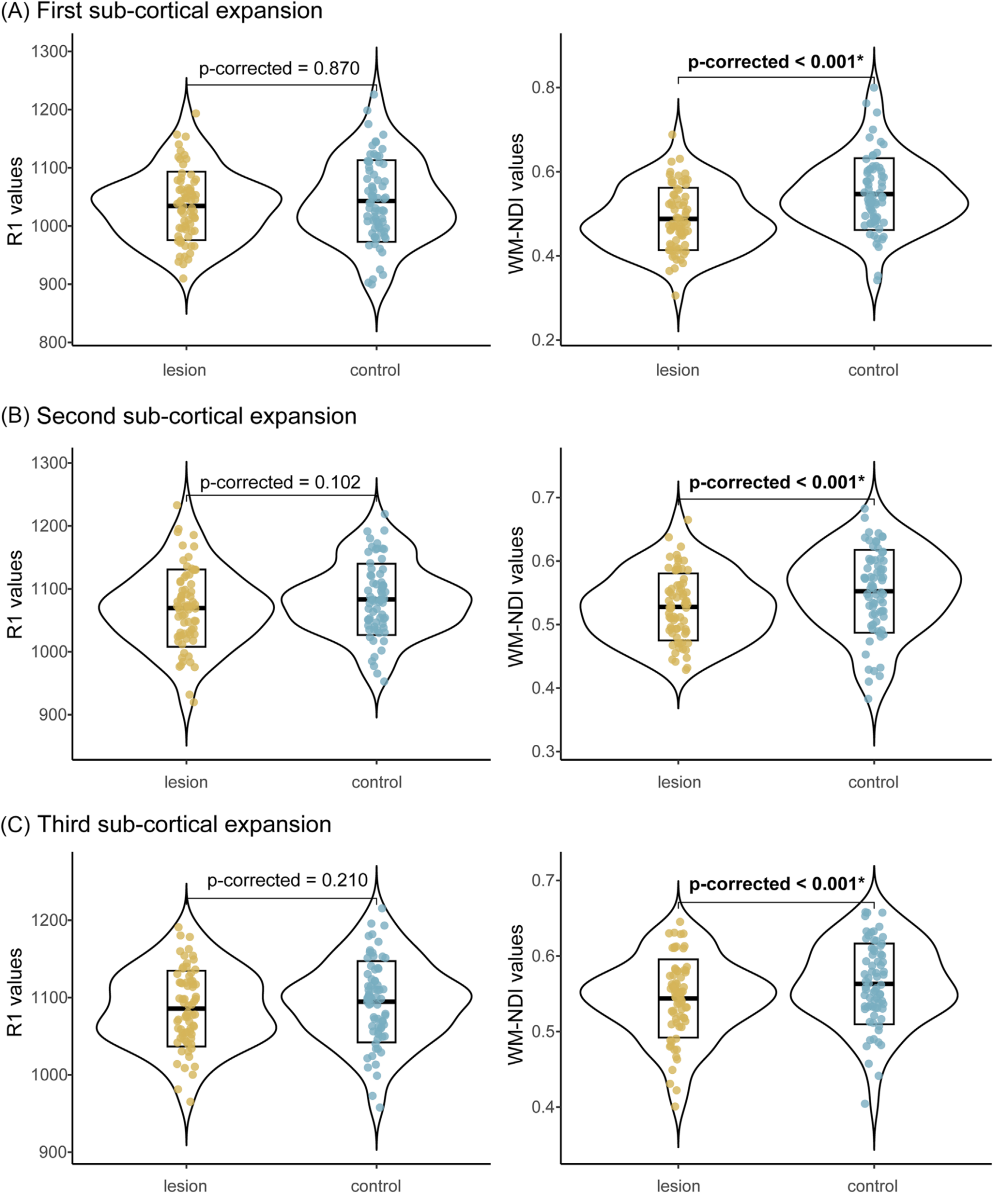
Comparisons of R1 and WM‐NDI values between old CMIs and control regions at each of their three sub‐cortical expansions. Group comparisons were conducted using paired‐sample *t*‐tests or Wilcoxon signed‐rank tests (denoted by “+”), as appropriate. (A) First sub‐cortical expansion, old CMIs showed lower WM‐NDI values compared to control regions. (B) Second sub‐cortical expansion, old CMIs showed lower WM‐NDI values compared to control regions. (C) Third sub‐cortical expansion, old CMIs showed lower WM‐NDI values compared to control regions. Significant differences (*p*‐corrected < 0.05) are indicated by an asterisk (*) and bold font. Among the 81 identified old CMIs, seven lesions located too close to the pial surface and three lesions with poor cortical expansion characteristics were excluded, resulting in a total of 71 old CMIs were included in the analysis. CMI, cortical cerebral microinfarct; WM‐NDI, white matter neurite density index.

**FIGURE 5:**
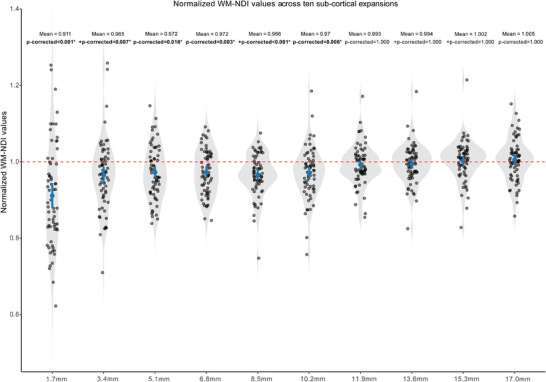
Normalized WM‐NDI values of old CMIs across 10 sub‐cortical expansions. Group comparisons were conducted using one‐sample *t*‐tests or Wilcoxon signed‐rank tests (denoted by “+”), as appropriate. Normalized WM‐NDI values within the first six subcortical expansions (10.2 mm) were lower than 1. Significant differences (*p*‐corrected < 0.05) are indicated by an asterisk (*) and bold font. CMI, cortical cerebral microinfarct; WM‐NDI, white matter neurite density index.

For recent CMIs, no MRI measure differences were found between lesion and control regions at any subcortical expansion (Figure S3). For old CMIs, WM‐NDI values were consistently lower at all three initial lesion expansions compared to corresponding control regions, whereas R1 values showed no significant differences between lesion and control regions at any of the three expansions (Figure 4). In addition, in the extended subcortical expansions, normalized WM‐NDI values were significantly lower than 1 within the first six subcortical expansions (10.2 mm) (Figure 5). The linear mixed model showed significant effects of both expansion distance (*β* = 0.004 [sta, *p* = 0.003) and lesion volume (*β* = –0.004 ± 0.001, *p* = 0.016) on normalized WM‐NDI ratios, as well as a significant interaction (*β* = 0.001 ± 0.0002, *p* < 0.001).

### 3.3 Temporal effects of CMIs at the lesion site

**FIGURE 6:**
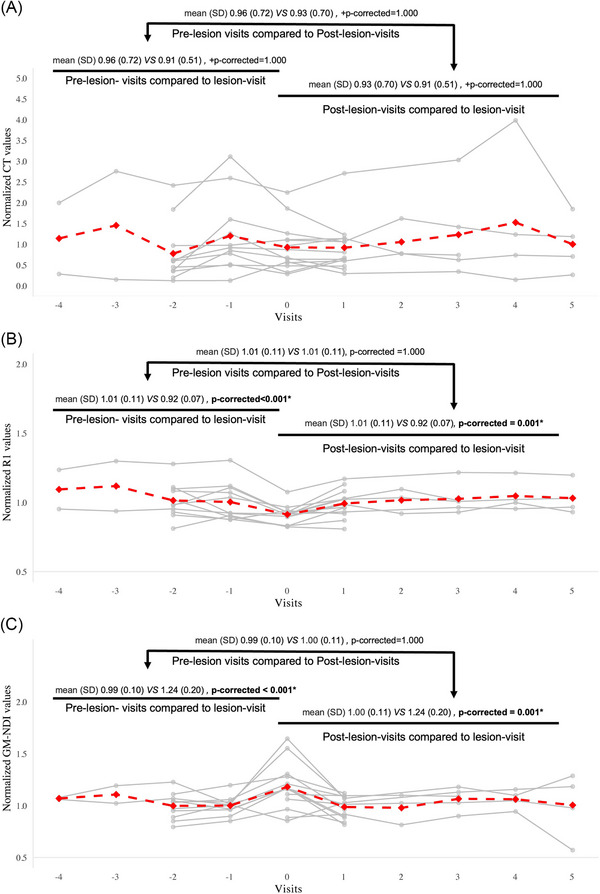
Comparison of normalized cortical thickness, R1, and GM‐NDI values of recent CMIs at the lesion site between pre‐lesion visits, lesion‐visit, and post‐lesion visits. Group comparisons were conducted using paired‐sample *t*‐tests or Wilcoxon signed‐rank tests (denoted by “+”), as appropriate. (A) recent CMIs showed no differences of the normalized CT values at the lesion site between pre‐lesion visits, lesion visit, and post‐lesion visits. (B) Recent CMIs showed lower normalized R1 values at the lesion site on the lesion visit compared to pre‐lesion visits and post‐lesion visits. (C) Recent CMIs showed higher normalized GM‐NDI values at the lesion site on the lesion visit compared to pre‐lesion visits and post‐lesion visits. Visit = 0 represents the lesion visit, that is, the visit when the lesion was identified; visit < 0 represents pre‐lesion visits, that is, visits prior to the lesion visit; and visit > 0 represents post‐lesion visits, that is, visits after the lesion‐visit. Significant differences (*p*‐corrected < 0.05) are indicated by an asterisk (*) and bold font. Among the 21 identified recent CMIs, two lesions located too close to the pial surface were excluded, resulting in a total of 19 recent CMIs included in the analysis. CMI, cortical cerebral microinfarct; CT, cortical thickness; GM‐NDI, gray matter neurite density index.

On the lesion visit, recent CMI sites showed significantly lower R1 values and higher GM‐NDI values compared to the control regions (Figure S4). Longitudinally, recent CMI sites showed lower R1 and higher GM‐NDI at the lesion visit compared to pre‐ and post‐lesion visits, with no differences between the latter two (Figure 6). In addition, normalized cortical thickness values showed no significant differences at lesion sites among three time points (Figure 6).

### 3.4 Sensitivity analysis

For old CMIs, MD values at the first sub‐cortical expansion were lower than those at the corresponding control regions, whereas no differences were found at the second and third sub‐cortical expansions (Figure S5).

## 4 DISCUSSION

In the present study, we found that (1) old CMIs were associated with lower WM‐NDI values in surrounding subcortical regions along the WM tracts, extending up to 10.2 mm; (2) longitudinally, recent CMIs showed lower R1 values and higher NDI values than their control regions at the time of lesion detection, which normalized during follow‐up. These findings suggest that old CMIs are linked to persistent sub‐cortical microstructural changes along descending WM tracts, suggesting a cortico‐subcortical (vertical) propagation of damage. In contrast, microstructural alterations associated with recent CMIs appear transient and localized to the lesion site.

We found that old CMIs were associated with reduced NDI values in both surrounding cortical and subcortical regions, with effects more pronounced in the subcortex (extending up to 10.2 mm subcortically vs 3.4 mm cortically). Because reduced NDI typically indicates reduced neurite density,^12^ these findings suggest secondary degeneration along WM tracts connected to old CMIs. This provides in vivo evidence that secondary neurodegeneration contributes to the non‐local effects of old CMIs and may underlie the global WM connectivity disruptions and cognitive impairment reported previously.^7^ Consistent with this interpretation, the linear mixed‐effects model showed that microstructural damage decreased with increasing distance from the lesion. Of interest, we observed a positive interaction effect between expansion distance and lesion size, indicating that larger CMIs exhibited a steeper spatial gradient of NDI alteration. Although unexpected, this finding offers new insight: small and large CMIs may induce degeneration over comparable spatial ranges, but larger lesions likely cause more severe tissue injury near the lesion core, resulting in greater distance‐dependent changes. This interpretation parallels previous studies on remote lesion effects, which showed that even less‐severe subcortical infarcts can induce cortical alterations comparable in magnitude to those caused by larger destructive lesions.^19^ By contrast, no similar differences were found for MD or R1 values across subcortical expansions. This may be because MD index from the DTI model is less specific to microstructure compared to the NDI index from the biophysical NODDI model.^12^ Furthermore, as WM is rich in myelin and R1 primarily reflects myelin, R1values may remain relatively stable in the presence of mild myelin damage.

Regarding cortical expansions of old CMIs, the extent of perilesional damage in our study was markedly smaller than that reported previously.^5^, ^6^ A previous study described cortical thinning extending up to 20 mm from CMI cores.^6^ In contrast, we only observed reduced NDI values limited to ≈3 mm from the lesion core, without cortical thinning. Given the diffusion image voxel size (1.7 mm) and the multiple co‐registration steps involved, these limited changes likely reflect partial volume effects (PVEs) or, at most, mild and spatially limited tissue damage. This discrepancy between cortical and subcortical alterations may be explained by cortical architecture: vertical fibers projecting to subcortex are much more abundant than horizontal intracortical fibers, forming the cortical columns.^20^ Consequently, secondary neurodegeneration may preferentially propagate along these vertical fibers, leading to more extensive subcortical than cortical damage. Of interest, higher R1 values were found in the first cortical expansion of old CMIs compared to control regions. This finding is unlikely to arise from PVEs, as R1 values at the lesion core itself were lower than in controls (Figure S6). Instead, elevated R1 values may reflect gliosis following tissue injury,^21^, ^22^ whereas increased macromolecular and glial content alters the molecular environment and the interaction between tissue bound water and macromolecules, leading to shortened T1 (1/R1).^23^ Further validation with high‐resolution MRI and histopathological studies are warranted.

Regarding the spatial effects of recent CMIs, we found higher NDI values in the first cortical expansion compared with control regions, likely reflecting PVE rather than perilesional damage. As suggested by previous histopathological studies, tissue injury of recent CMIs is relatively minor.^24^ In our study, recent CMIs exhibited higher NDI values and lower R1 values at the lesion site compared to control regions. The increased NDI values likely reflect neuronal swelling due to cytotoxic edema during the early phase after lesion onset.^25^, ^26^ Alternatively, the underlying assumptions of the NODDI model, such as fixed diffusivity and tortuosity constraints, may not fully hold in the acute infarct stage, potentially affecting NDI estimates.^27^ The reduction in R1 may indicate increased water content due to vasogenic edema or early demyelination, both of which enhance water mobility.

Longitudinally, both R1 and NDI values at recent CMI lesion sites normalized on follow‐up MRI scans. This is consistent with our previous findings that all recent CMIs became undetectable over time.^13^ These findings further support our speculation that observed R1 and NDI changes at the recent CMI lesion site reflect neuronal swelling transient edema rather than irreversible tissue damages. However, a previous study from our group suggested that increased R2* values at the recent CMIs sites persist over time.^28^ This may indicate that iron accumulation (as measured by increased R2*) may arise not from ongoing myelin or neurite disruption, but rather from increased cell membrane permeability or vascular leakage of blood cells.^29^

Notably, although the present study did not directly assess clinical outcomes, the distinct spatial and temporal patterns observed between recent and old CMIs offer mechanistic insights into how these lesions may contribute to the clinical manifestations of SVD. Specifically, secondary degeneration along connected WM tracts could represent a crucial pathway through which old CMIs disrupt WM integrity and structural connectivity, ultimately leading to cognitive impairment.^4^, ^7^, ^8^, ^30^, ^31^ In contrast, the transient alterations in R1 and NDI observed in recent CMIs likely reflect acute edema and cellular swelling that resolve without permanent structural damage. Nevertheless, the cumulative burden of cortical microvascular pathology remains an important consideration,^31^ as the presence of visible cortical CMIs may signify numerous microscopic cortical infarcts that remain undetectable on conventional MRI. Further studies are warranted to determine the clinical relevance of these recent CMIs and their contribution to disease progression.

The strengths of this study include a high‐frequency serial imaging design using state‐of‐the‐art multimodal neuroimaging. Monthly MRI scans allowed us to capture acute events and characterize their dynamics in the short‐term. Some limitations should be acknowledged. First, MRI‐derived indices such as R1 and NDI are indirect measures of tissue properties; thus, the underlying histological features can only be inferred. The small size of CMIs further increases the potential impact of registration inaccuracies and PVEs. To minimize these confounds, we have taken several measures. Lesions located too close to the pial surface were excluded from the analysis to reduce cerebrospinal fluid (CSF)–related confounding signal. All MRI indices were calculated in each individual native volume space to avoid unnecessary resampling. When spatial normalization was required, we used the state‐of‐the‐art registration algorithm implemented in Advanced Normalization Tools (ANTs), followed by visual inspection and manual correction, ensuring precise alignment of lesion and control regions. Furthermore, in interpreting our results, we explicitly considered potential PVE contributions. Nonetheless, future studies employing high‐resolution MRI and histopathological studies are warranted to confirm the underlying biological interpretation of our findings. Second, the temporal resolution of our imaging protocol limits the interpretation of CMI‐related damage evolution. All old CMIs were already present at baseline and no additional old lesions appeared during follow‐up. Thus, associations between old CMIs and subcortical WM damage could only be examined cross‐sectionally. Recent CMIs were newly detected at a single visit and disappeared on subsequent imaging ≈1 month later. Consequently, our observations were restricted to monthly intervals, thereby limiting the ability to capture more rapid dynamic changes. Future longitudinal studies with longer follow‐up for old CMIs and more frequent imaging intervals (e.g., weekly) for recent CMIs are needed to delineate the temporal relationship between CMI onset, duration, and subsequent tissue damage. Finally, a limitation is the relatively small number of CMIs, particularly recent CMIs (*n* = 19), included in the analysis. This constraint arises from the inherently transient nature of DWI‐visible CMIs, which appear only within a narrow temporal window of diffusion restriction. Despite intensive longitudinal MRI follow‐up (472 DWI scans in 54 participants over 10 months), only 21 recent CMIs were detected across 7 participants. However, our lesion‐based analytical design, in which each lesion was compared with its contralateral control region, and our targeted recruitment of individuals with SVD, while excluding other possible pathologies, minimized inter‐individual variability and increased statistical sensitivity. Notably, recent experimental evidence from animal models demonstrates that microinfarcts induce transient neural suppression with minimal cell death,^32^ paralleling the temporary, localized alterations observed in our study. Nevertheless, future large‐scale, high‐frequency imaging studies are warranted to validate and extend these findings.

In conclusion, the present study provided a comprehensive assessment on the temporal and spatial effects of CMI‐related damage using high‐frequency serial multi‐modal images. Our findings showed that microstructural abnormalities from recent CMIs are confined largely to lesion site and tend to be transient. By contrast, old CMIs are associated with sub‐cortical damage along WM tracts but are not likely to affect adjacent cortical regions. These findings clarified the distinct spatial and temporal effects of recent versus old CMIs on cortical and subcortical microstructural changes, thereby advancing our understanding of CMIs and their impact on brain structure, potentially explaining the mechanisms of cognitive impairment in SVD.

## CONFLICT OF INTEREST STATEMENT

The authors declare no conflicts of interest. Author disclosures are available in the supporting information.

## CONSENT STATEMENT

All participants provided written informed consent, and the study was approved by the Medical Review Ethics Committee of the Region Arnhem‐Nijmegen.

## Supporting information



Supporting Information

Supporting Information
