# Supplementary material for: Spatial and temporal effects of cortical cerebral microinfarcts on the cortical and subcortical regions in cerebral small vessel disease
Source: Alzheimers Dement. 2025 Dec 29;21(12):e71056. doi: 10.1002/alz.71056 (PMC12746041; doi:10.1002/alz.71056)
Supplement: Supplementary file 1 — Supporting Information [file ALZ-21-e71056-s001.docx]

# **Supplementary materials**

# **Materials and methods**

## **Quantitative MRI measures: cortical thickness, R1 and Neurite Density Index**

For cortical thickness (CT), we first used a homemade MATLAB (R2016b; MathWorks, Natick, MA) script to create the robust T1W image based on MP2RAGE sequence.^1^ Subsequently, we performed this volumetric CT calculation on the robust T1W image using DL + DiReCT, a tool based on diffeomorphic registration-based CT measurement.^2^ This approach has demonstrated accuracy and reliability in CT measurements, comparable to those obtained from surface-based methods using FreeSurfer.^3^

For R1, MP2RAGE images were processed to obtain the quantitative T1 map using an in-house MATLAB script.^4^ The quantitative R1 map was created by taking the reciprocal of the T1 map and scaling that in mHz (ms^−1^). The R1 map and the robust T1 image are intrinsically aligned since they are from the same data (i.e. MP2RAGE image).

For Neurite Density Index (NDI), we firstly preprocessed the multi-shell diffusion MRI data to denoise and remove Gibbs artifact, correct head motion and eddy current-induced distortions, mitigate susceptibility-induced distortions (topup) and intensity bias using the Functional Magnetic Resonance Imaging of the Brain Software Library (FSL; v6.0.1) and the Advanced Normalization Tools (ANTs, v 2.1.0).^5-9^ The parameter of neurite compartment intrinsic parallel diffusivity [d∥] within the neurite orientation dispersion and density imaging (NODDI) model differs between white matter (WM) and grey matter (GM).^10^ Consequently, when applying the NODDI model to WM, we set the d∥ value at 1.7 µm2/ms, which has been fixed using the corpus callosum as a reference, yielding the WM-NDI map for each participant; when employing NODDI model in the GM, we set its value to 1.2 µm2/ms, which is in the range of values proposed to minimise model residual for brain GM, yielding the GM-NDI map for each participant.^10^

## **Lesion expansion for cortical micro-infracts**

For cortical expansion of recent CMIs and their control regions, these ROIs were registered from DWI space to T1W and then expanded six times in T1W space, each time increasing by one voxel (voxel size: 0.85 mm). To ensure these expanded regions were confined to the cortex, a cortical segmentation mask was applied during each expansion. Next, the ROIs and their three cortical expansions at 1.7 mm, 3.4 mm, and 5.1 mm in DWI space were registered back to T1W space. Regarding the subcortical expansion, probabilistic streamlines tractography using multi-shell DWI image were firstly performed to capture the WM tracts passing through the ROIs.^11,12^ These ROIs in DWI space were expanded three times (1.7 mm, 3.4 mm, and 5.1 mm), each time increasing by one voxel (1.7 mm). Each expansion was restricted to the sub-cortical section of WM tracts connecting these ROIs by using a combination of WM and the corresponding tract-specific masks. The ROIs and their sub-cortical expansions were then registered to T1W space. The transformation between different modality within subject were performed using ANTs software.^13^

For cortical expansion of old CMIs and their control regions, ROIs in T1W space were incrementally expanded six times, each by one voxel (voxel size: 0.85 mm), with expansions confined to the cortical region using a cortical segmentation mask. The ROIs and their expansions at 1.7 mm, 3.4 mm, and 5.1 mm in T1 space were then registered to T1W space using ANTs software.^13^ Regarding the sub-cortical expansions, these ROIs in T1W space were registered to DWI space using the ANTs software.^13^ Subsequently, the sub-cortical sections of tracts passing through these ROIs in DWI space and their expansions were identified following the same methodology as with recent CMIs. These ROIs along with their expansions at 1.7 mm, 3.4 mm, 5.1 mm, were registered to DWI space using ANTs software.^13^

For probabilistic streamlines tractography, fiber orientation distributions (FOD) images were estimated using the multi-tissue constrained spherical deconvolution (MT-CSD) algorithm in MRtrix 3.0 software.^11,14^ These FOD images were then used to track fibers passing through the recent CMI lesions or control regions, employing these regions as seed masks and the second-order integration over fiber orientation distributions (iFOD2) algorithm.^12^

## **Extraction of quantitative MRI measures**

All NDI maps from each visit were initially aligned with the corresponding T1W images of the same visit using using “antsRegistrationSyNQuick” function within ANTs software.^13^ Subsequently, all T1W images from different visits were registered to the T1W image from the visit during which the lesion was identified using “antsRegistrationSyN” function.^13^ Transformation matrices produced in this second registration step were then used to align the CT, R1, and GM-NDI maps from pre-lesion and post-lesion visits with the T1W image from the lesion-visit.

# **Reference**

1. Marques JP, Kober T, Krueger G, van der Zwaag W, Van de Moortele PF, Gruetter R. MP2RAGE, a self bias-field corrected sequence for improved segmentation and T1-mapping at high field. Neuroimage. Jan 15 2010;49(2):1271-81. doi:10.1016/j.neuroimage.2009.10.002

2. Rebsamen M, Rummel C, Reyes M, Wiest R, McKinley R. Direct cortical thickness estimation using deep learning-based anatomy segmentation and cortex parcellation. Hum Brain Mapp. Dec 2020;41(17):4804-4814. doi:10.1002/hbm.25159

3. Das SR, Avants BB, Grossman M, Gee JC. Registration based cortical thickness measurement. Neuroimage. Apr 15 2009;45(3):867-79. doi:10.1016/j.neuroimage.2008.12.016

4. Shams Z, Norris DG, Marques JP. A comparison of in vivo MRI based cortical myelin mapping using T1w/T2w and R1 mapping at 3T. PLoS One. 2019;14(7):e0218089. doi:10.1371/journal.pone.0218089

5. Kellner E, Dhital B, Kiselev VG, Reisert M. Gibbs‐ringing artifact removal based on local subvoxel‐shifts. Magnetic resonance in medicine. 2016;76(5):1574-1581.

6. Veraart J, Novikov DS, Christiaens D, Ades-Aron B, Sijbers J, Fieremans E. Denoising of diffusion MRI using random matrix theory. Neuroimage. 2016;142:394-406.

7. Smith SM, Jenkinson M, Woolrich MW, et al. Advances in functional and structural MR image analysis and implementation as FSL. Neuroimage. 2004;23:S208-S219.

8. Andersson JL, Sotiropoulos SN. An integrated approach to correction for off-resonance effects and subject movement in diffusion MR imaging. Neuroimage. 2016;125:1063-1078.

9. Tustison NJ, Avants BB, Cook PA, et al. N4ITK: improved N3 bias correction. IEEE transactions on medical imaging. 2010;29(6):1310-1320.

10. Guerrero JM, Adluru N, Bendlin BB, et al. Optimizing the intrinsic parallel diffusivity in NODDI: An extensive empirical evaluation. PLoS One. 2019;14(9):e0217118. doi:10.1371/journal.pone.0217118

11. Tournier JD, Smith R, Raffelt D, et al. MRtrix3: A fast, flexible and open software framework for medical image processing and visualisation. Neuroimage. Nov 15 2019;202:116137. doi:10.1016/j.neuroimage.2019.116137

12. Tournier JD, Calamante F, Connelly A. Improved probabilistic streamlines tractography by 2nd order integration over fibre orientation distributions. John Wiley & Sons, Inc New Jersey, NJ; 2010:

13. Avants BB, Tustison NJ, Song G, Cook PA, Klein A, Gee JC. A reproducible evaluation of ANTs similarity metric performance in brain image registration. Neuroimage. Feb 1 2011;54(3):2033-44. doi:10.1016/j.neuroimage.2010.09.025

14. Jeurissen B, Tournier JD, Dhollander T, Connelly A, Sijbers J. Multi-tissue constrained spherical deconvolution for improved analysis of multi-shell diffusion MRI data. Neuroimage. Dec 2014;103:411-426. doi:10.1016/j.neuroimage.2014.07.061


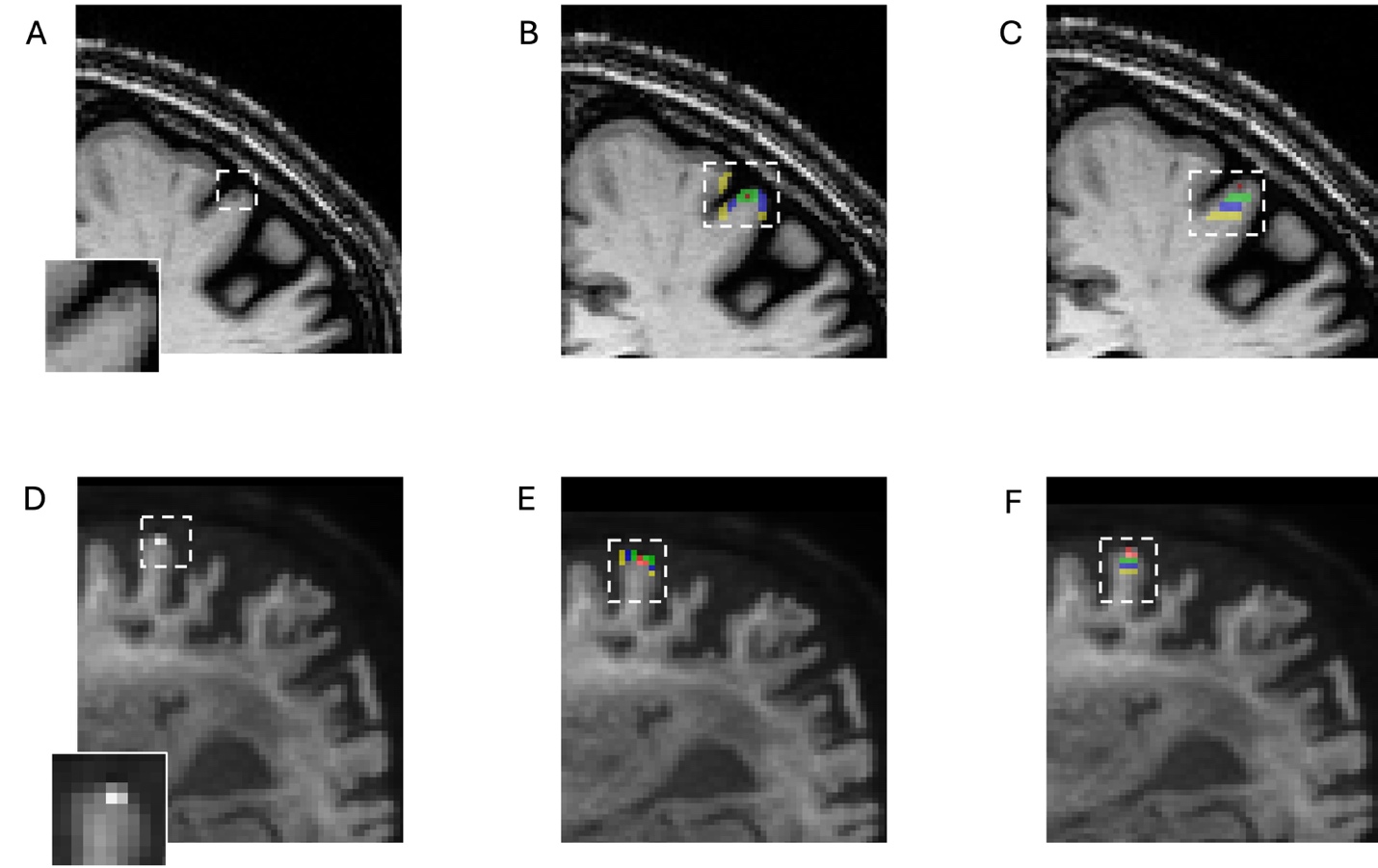


Figure e-1. Example images of recent and old CMIs and their cortical and sub-cortical expansions. A. old CMI; B, cortical expansions of old CMI; C, sub-cortical expansions of old CMI; D. recent CMI; E, cortical expansions of recent CMI; F, sub-cortical expansions of recent CMI. Red: original lesion site for recent or old CMIs; green: first expansion at 1.7mm; blue: second expansion at 3.4mm; yellow: third expansion at 5.1mm.


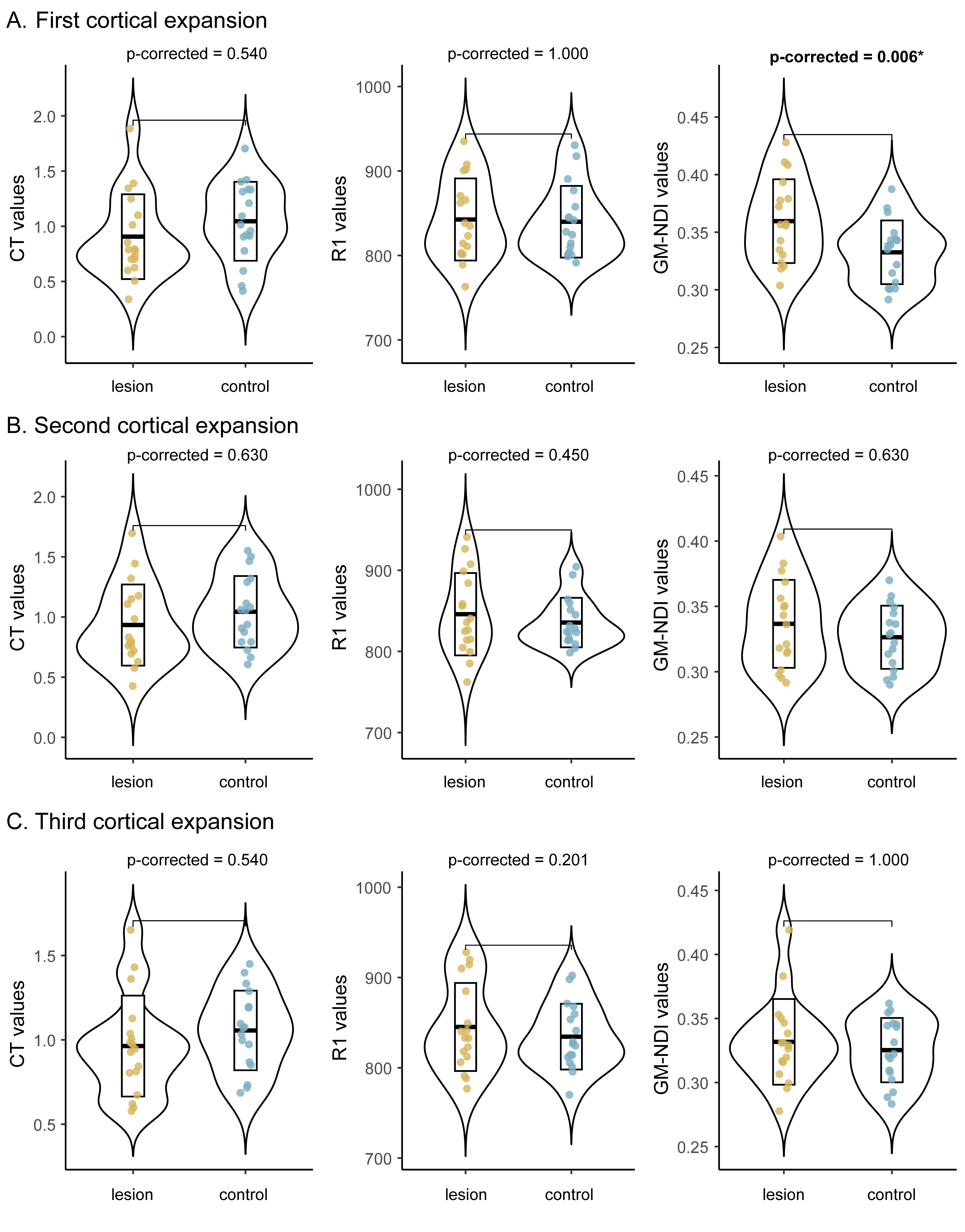


Figure e-2. Comparisons of CT, R1 and GM-NDI values between recent CMIs and control regions at each of their three cortical expansions. Group comparisons were conducted using paired-sample t-tests or Wilcoxon signed-rank tests (denoted by “+”), as appropriate. A. First cortical expansion, recent CMIs showed higher NDI values compared to control regions; B and C. Second and third cortical expansion, recent CMIs showed no differences at these MRI measures compared to control regions. CT, cortical thickness, GM-NDI, neurite density index for grey matter. Significant differences (p-corrected < 0.05) are indicated by an asterisk (*) and bold font. Among the 21 identified recent CMIs, 2 lesions located too close to the pial surface and 2 lesions with poor cortical expansion characteristics were excluded, resulting in a total of 17 recent CMIs included in the analysis.


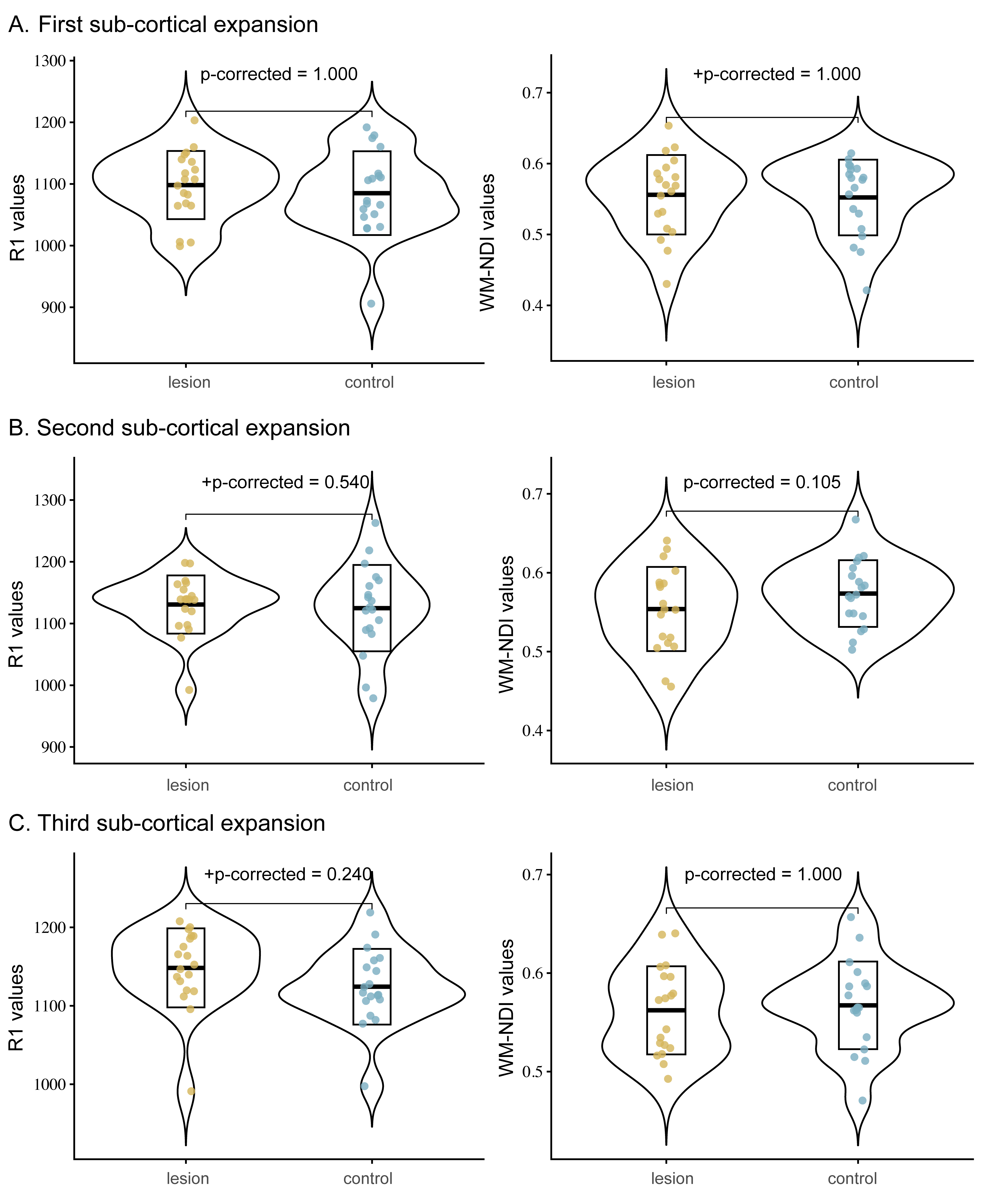


Figure e-3. Comparisons of CT, R1 and WM-NDI values between recent CMIs and control regions at each of their three sub-cortical expansions. Group comparisons were conducted using paired-sample t-tests or Wilcoxon signed-rank tests (denoted by “+”), as appropriate. All three sub-cortical expansion showed no differences at these MRI measures compared to control regions. CT, cortical thickness; WM-NDI, neurite density index for white matter. For recent CMIs, 21 lesions were identified, two lesions located too close to the pial surface were excluded, resulting in a total of 19 lesions included in the analysis.


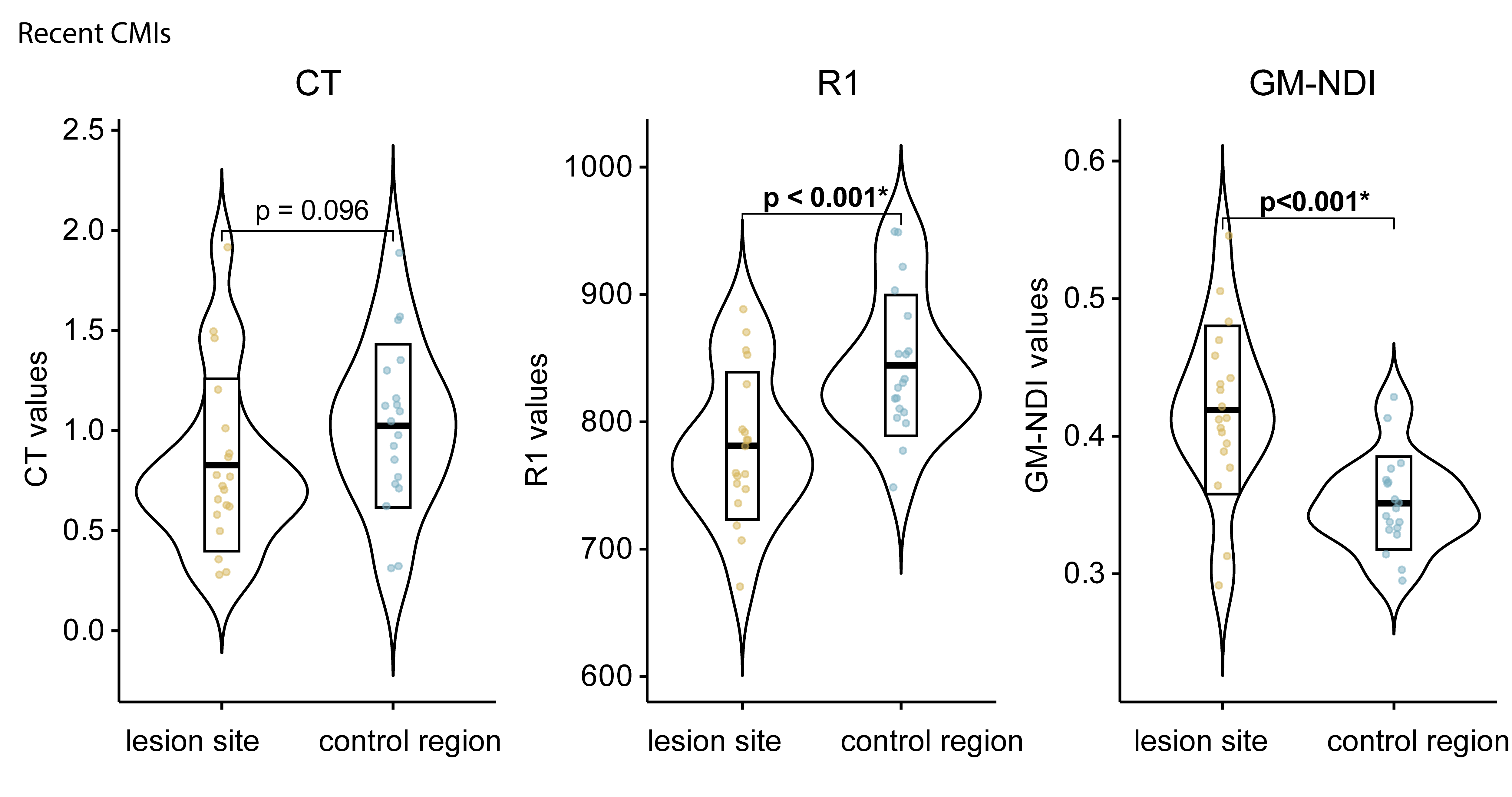


Figure e-4. Comparison of the cortical thickness, R1 and NDI values between the lesion site of recent CMIs and control regions. Group comparisons were conducted using paired-sample t-tests or Wilcoxon signed-rank tests (denoted by “+”), as appropriate. At the lesion site, recent CMIs showed lower R1, and higher NDI values compared to control regions. Significant differences (p < 0.05) are indicated by an asterisk (*) and bold font. Among the 21 recent CMIs identified, 2 located too close to the pial surface were excluded due to potential confounding from partial volume effects, resulting a total of 19 recent CMIs were included in the analysis.


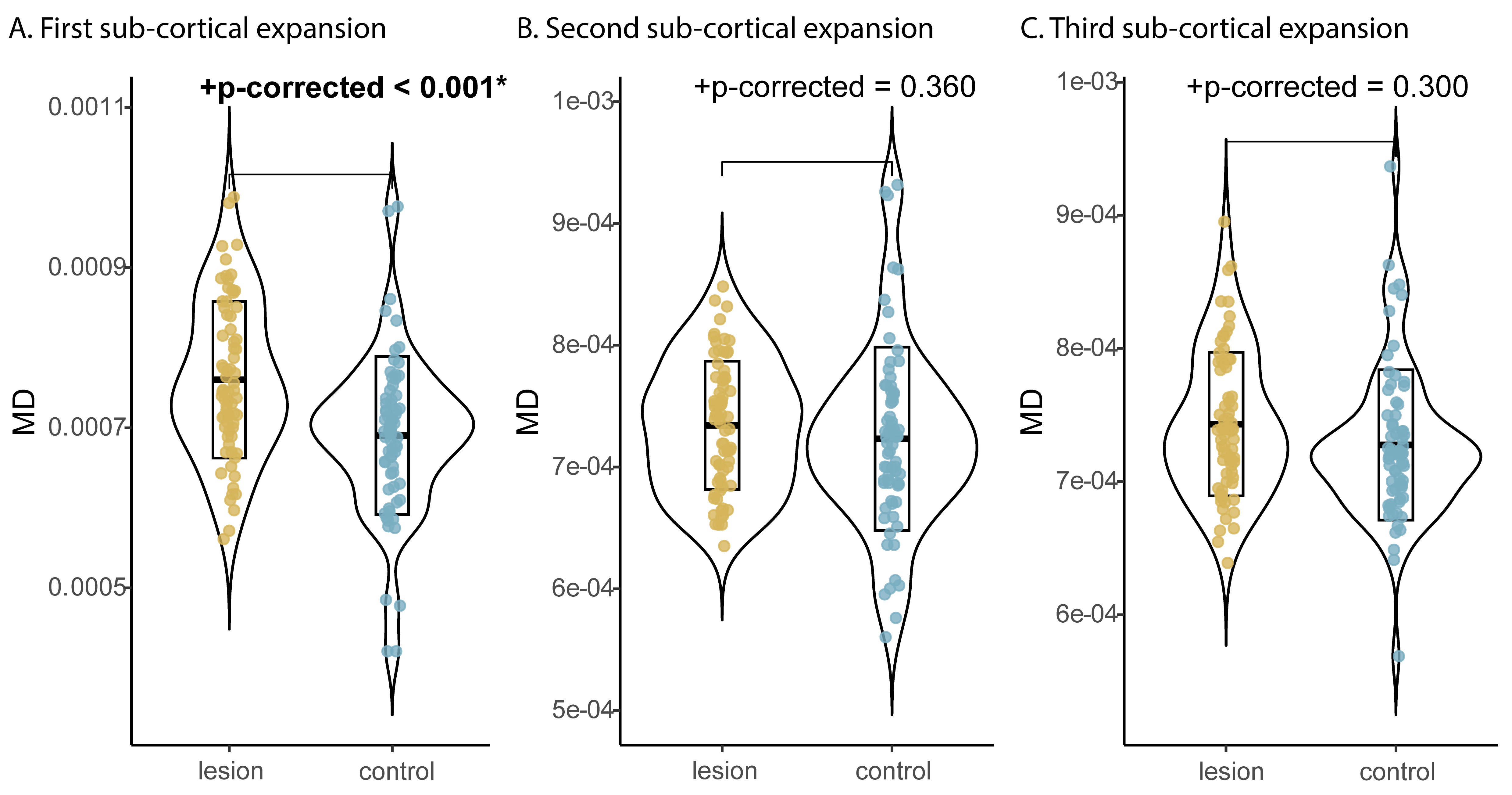


Figure e-5. Comparison of MD values between old CMIs and control regions at each of their three sub-cortical expansions. Group comparisons were conducted using paired-sample t-tests or Wilcoxon signed-rank tests (denoted by “+”), as appropriate. MD values at the first expansion were significantly lower than those at the corresponding control regions. MD, mean diffusivity, Significant differences (p-corrected < 0.05) are indicated by an asterisk (*) and bold font. Among the 81 identified old CMIs, 7 lesions located too close to the pial surface and 3 lesions with poor cortical expansion characteristics were excluded, resulting in a total of 71 old CMIs were included in the analysis.


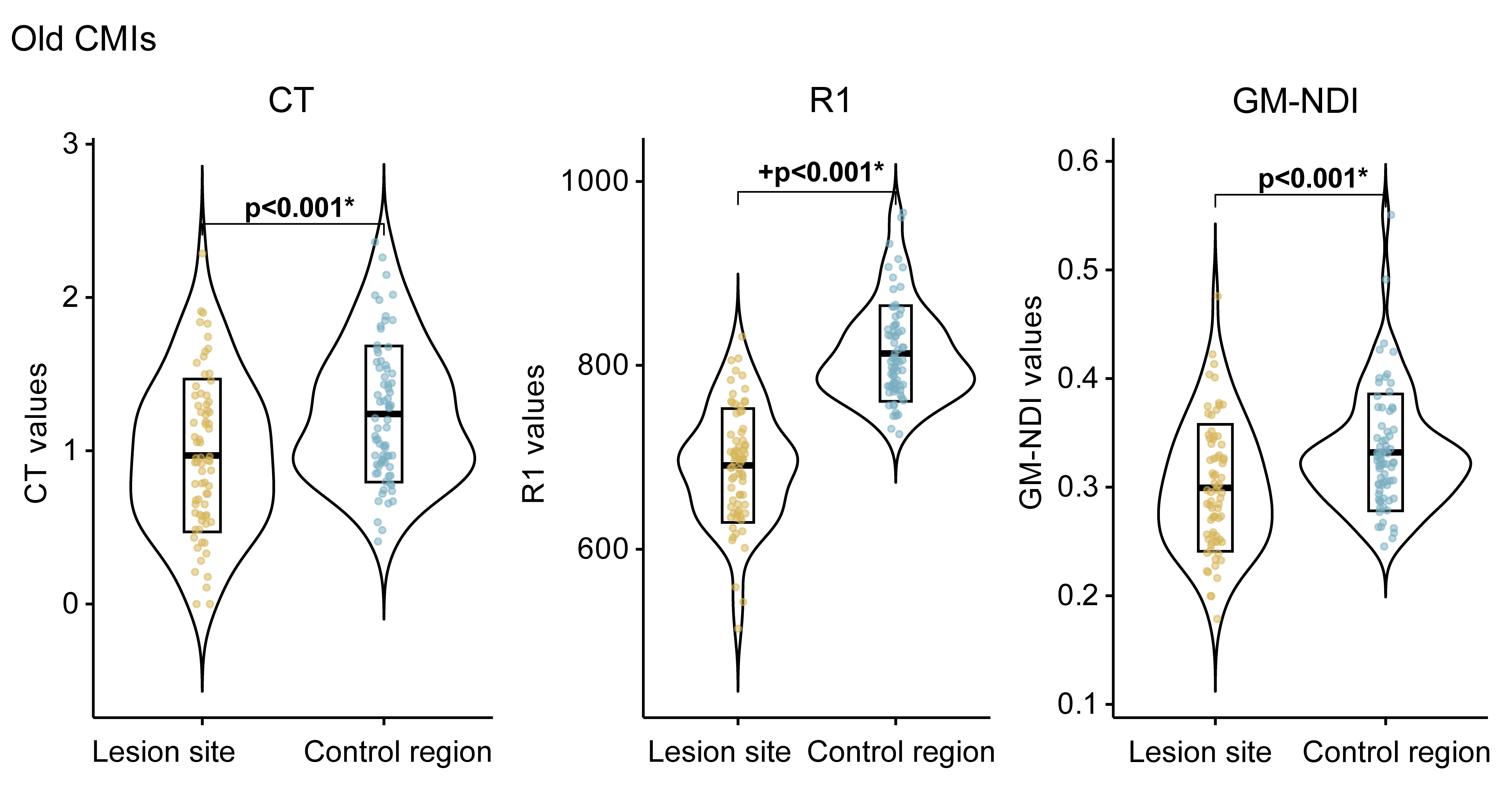


Figure e-6. Comparison of the cortical thickness, R1 and NDI values between the lesion site of old CMIs and control regions. Group comparisons were conducted using paired-sample t-tests or Wilcoxon signed-rank tests (denoted by “+”), as appropriate. At the lesion site, old CMIs showed lower CT, lower R1, and lower GM-NDI values compared to control regions. Significant differences (p < 0.05) are indicated by an asterisk (*) and bold font. Among the 81 old CMIs identified, 7 lesions located too close to the pial surface were excluded due to potential confounding from partial volume effects, resulting a total of 74 old CMIs were included in the analysis.
